# RADAI: A Deep Learning-Based Classification of Lung Abnormalities in Chest X-Rays

**DOI:** 10.3390/diagnostics15131728

**Published:** 2025-07-07

**Authors:** Hanan Aljuaid, Hessa Albalahad, Walaa Alshuaibi, Shahad Almutairi, Tahani Hamad Aljohani, Nazar Hussain, Farah Mohammad

**Affiliations:** 1 Computer Science Department, College of Computer and Information Science, Princess Nourah Bint Abdulrahman University, Riyadh 11671, Saudi Arabia; 2 Research Chair of AI in Healthcare, Princess Nourah Bint Abdulrahman University, Riyadh 11564, Saudi Arabia; hassea2020@gmail.com (H.A.); walaaalshuaibo@gmail.com (W.A.); shahadalakshan34@gmail.com (S.A.); thaljohani@pnu.edu.sa (T.H.A.); fnazar@ieee.org (F.M.); 3 Information Technology Department, College of Computer and Information Sciences, Princess Nourah Bint Abdulrahman University, Riyadh 11564, Saudi Arabia

**Keywords:** diagnose, chest X-ray, deep learning, image classification, convolutional neural networks (CNNs), King Abdullah University Hospital (KAAUH)

## Abstract

**Background:** Chest X-rays are rapidly gaining prominence as a prevalent diagnostic tool, as recognized by the World Health Organization (WHO). However, interpreting chest X-rays can be demanding and time-consuming, even for experienced radiologists, leading to potential misinterpretations and delays in treatment. **Method:** The purpose of this research is the development of a RadAI model. The RadAI model can accurately detect four types of lung abnormalities in chest X-rays and generate a report on each identified abnormality. Moreover, deep learning algorithms, particularly convolutional neural networks (CNNs), have demonstrated remarkable potential in automating medical image analysis, including chest X-rays. This work addresses the challenge of chest X-ray interpretation by fine tuning the following three advanced deep learning models: Feature-selective and Spatial Receptive Fields Network (FSRFNet50), ResNext50, and ResNet50. These models are compared based on accuracy, precision, recall, and F1-score. **Results:** The outstanding performance of RadAI shows its potential to assist radiologists to interpret the detected chest abnormalities accurately. **Conclusions:** RadAI is beneficial in enhancing the accuracy and efficiency of chest X-ray interpretation, ultimately supporting the timely and reliable diagnosis of lung abnormalities.

## 1. Introduction

Chest X-rays are the most widely used medical imaging technique for diagnosing lung abnormalities [1]. Despite the emergence of new sophisticated techniques, chest X-rays remain critical as they are usually the first diagnostic procedures recommended by doctors [2]. Moreover, about 70 million chest X-rays are conducted annually in the US alone [3]—reading and interpreting chest X-rays is still challenging. Medical technicians’ study for approximately five years in radiology to understand X-rays accurately [4]. In recent years, a new era of deep learning models has emerged leading to CNNs specialized in analyzing and classifying images [5]. It did not take long before it broke through into the medical field to automate the process of reading chest X-rays. Many researchers developed deep learning- or CNN-based models for X-ray image analysis and compared the results with practicing radiologists, showing the importance of deep learning models over manual analysis [6].

Despite significant efficiency, one of the issues with CNNs includes the requirement for large annotated datasets [7,8]. To annotate X-ray images, a trained expert must go through the images and annotate them manually, making the process of finding a good expert and waiting for them to annotate images time-consuming. Therefore, utilizing an open-source NIH chest X-ray 14 dataset [9], an annotated dataset which has been approved by radiologists worldwide, is much more convenient. Another issue arises when encountering class imbalance, especially in multi-class classification tasks where samples of some classes are in the minority compared to others [10]. The issue of class imbalance needs to be handled very carefully to achieve meaningful results from CNN models. A recent study [11] on CNN-based chest X-ray abnormalities reported the highest area under the receiver operating characteristic curve (AUC) for hernia and emphysema as 0.9450 and 0.9120, respectively, out of 14 different categories under discussion. This is due to the poor availability of data according to abnormalities. Moreover, in medicine it is known that each disease has a different prevalence in the population [12]. For example, after being declared a pandemic in 2020, COVID-19 has garnered a significant amount of registered data, while tuberculosis, on the other hand, is not as prevalent in terms of data size [13]. An AI algorithm on chest X-ray analysis [14] could not perform better because of data limitations. Therefore, understanding the limitations associated with the amount of data is important. This study utilizes a ResNet50-based CNN model that leverages residual connections to enable the effective training of deep networks by mitigating the vanishing gradient problem. These connections facilitate the direct flow of information across layers, allowing the model to maintain performance as depth increases. ResNet50 demonstrates strong generalization and achieves high accuracy across a range of computer vision tasks, including medical images [15]. It is readily accessible through pre-trained models in major deep learning frameworks such as Keras and PyTorch, supporting the ease of integration and fine-tuning. Compared to architectures like VGGNet and Inception, ResNet50 offers improved depth handling and competitive performance [16]. While newer models like EfficientNet have pushed accuracy boundaries, ResNet50 remains an efficient and reliable choice, particularly in scenarios with limited computational resources. Moreover, the multiple variants of ResNet50 have been developed such as ResNext50 [17] and ResNet50 [18] to improve its performance. Therefore, in this study, a comparative analysis of ResNet50 and its two variants has been provided using chest X-ray images.

CNN algorithms are often considered ‘black box’ algorithms because end-users can only analyze the input and output labels [19]; the inference process is unclear, reducing confidence in these algorithms. Thus, explainable AI techniques, such as heatmaps [20], have also been developed to address this issue. Heatmaps highlight the pixels with the most influence on the final prediction. An early detection of this problem is crucial in radiography as it enables radiologists to detect errors in the diagnosis, ensuring that no errors reach the patient.

One of the challenges which the field of radiography faces is subjectivity. It is worth noting that the final diagnosis depends on the radiologist and their expertise [21]. For instance, a radiologist who commonly deals with lung-related cases may need to be more familiar with diagnosing heart conditions and is more likely to misdiagnose. Additionally, local radiologists may face challenges in diagnosing rare or unfamiliar diseases in their area. Given these challenges, developing a standardized diagnostic system is of paramount importance. An automated system could help to reduce the workload on radiologists and provide more consistent and accurate diagnosis, leading to better patient care and related outcomes.

This paper contributes to medical image analysis and automation which not only improves efficiency of the results but also reduces the repercussions of inaccuracies in results, directly benefiting the population of Saudi Arabia and beyond. The interpretation of chest X-ray images is a challenging and time-consuming process and may lead to possible misinterpretations and treatment delays, which in some cases is more than just trivial and can be life-threatening. Therefore, it seeks to both lighten the radiologist’s burden as well as minimize diagnosis subjectivity by creating a deep learning model, termed RadAI, that can accurately detect and classify any abnormal findings in the chest X-ray images. The specific objectives of this study include:

1. The study compares and evaluates three deep learning models, e.g., FSRFNet50 [22], ResNext50 [17], and ResNet50 [18], for the purpose of classifying four different abnormalities along with normal chest X-ray images.
2. RadAI leverages variants of the ResNet architecture to effectively capture complex features, which is essential for detecting abnormalities in chest X-ray images with high accuracy.
3. Additionally, a preprocessing step is employed to remove the diaphragm region in the chest X-ray—often a source of noise for deep learning models—enhancing both accuracy and training efficiency. Beyond chest X-ray abnormality detection, RadAI shows promise for broader applications in medical imaging tasks.
4. Also, this study highlights the potential of the proposed RadAI system for integration into clinical diagnostic workflows, aiming to support radiologists in making faster and more accurate diagnoses.

It is important to note that the system’s scope is limited to the classification of chest X-ray abnormalities and does not extend to treatment recommendations or clinical decision-making. The system functions as a diagnostic tool, aiding medical professionals in identifying abnormalities and enabling informed decision-making based on the classification results. Furthermore, the system does not explore other X-rays; its focus is solely on the abnormalities in the chest area.

## 2. Materials and Methods

To identify and define the primary requirements of our project, an interview was conducted with a radiologist from KAAUH, Riyadh, Kingdome of Saudi Arabia. During this interview, we focused on gathering insights into the hospital’s old system, which has been used for a short time and which we aim to replace with improvements. The radiologist shared valuable information that was unanimously believed regarding the old system, including the outstanding features and the limitations. We recorded their valuable observations and summarized them.

Our findings revealed that one of the key features of the old system was its seamless integration with the X-ray machine. As soon as an X-ray image was generated, it displayed both the original image and a colorful highlighted version. The colorful version of the X-ray image was being generated with the help of integrated AI to show the imperative regions. This dual representation allowed radiologists to quickly assess the areas of concern only. However, a significant challenge surfaced during the discussion. The radiologists expressed concerns about the system’s accuracy, as it was evident that they could not solely rely on the system’s assessments and often had to re-evaluate each image manually. Despite this, they acknowledged that the highlighted image did aid in detecting potential regions that might have otherwise been overlooked. This information gathered from the interview serves as a crucial foundation for our project scope, guiding our efforts to create a more efficient and dependable system for the radiologists at KAAUH.

The project’s scope lies in designing a deep learning-based system for classifying and identifying chest X-ray abnormalities and developing a user-friendly interface for easy access and usage. The primary goal is to accurately and efficiently categorize chest X-ray images into different abnormality categories, assisting medical professionals in diagnosing and interpreting.

**Figure 1:**
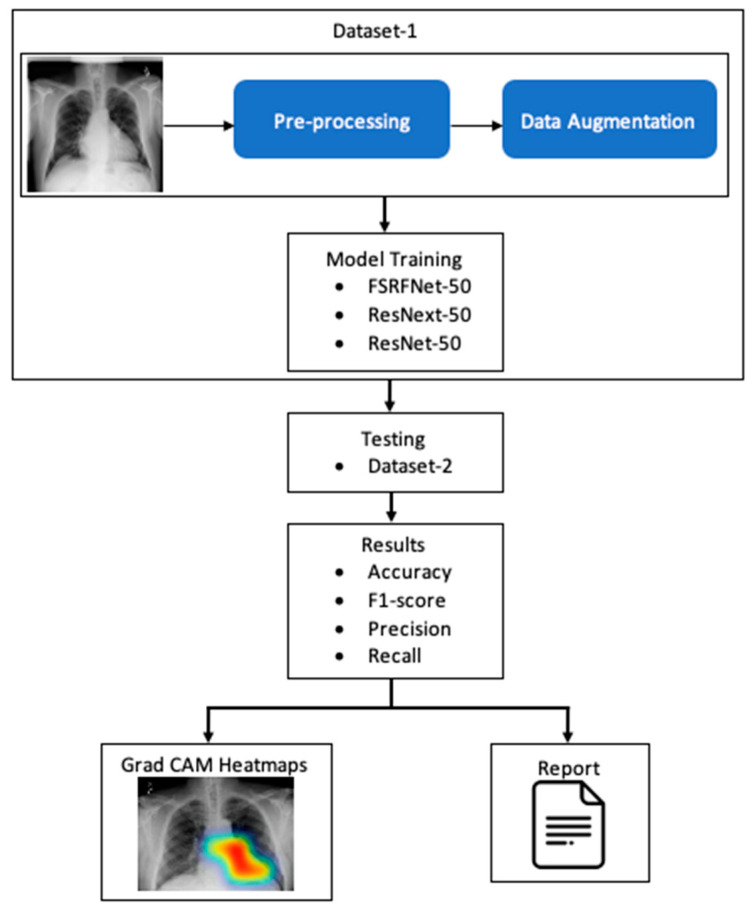
A simple conceptualization of RadAI system.

The RadAI system (Figure 1) involves preprocessing of the X-ray image to enhance its quality. The processed images are then used for training the deep learning model. The classifiers are then tested using different datasets to make the model unbiased. The tested image goes through a heatmap generation process, highlighting the regions of interest related to abnormality. Moreover, the same results are also fed into the report generation module to generate a simple clinical report about detected abnormalities.

### 2.1. Dataset and Augmentation

We obtained the KAAUH dataset from King Abdullah bin Abdulaziz University Hospital. The dataset contains only the following four distinct labels: cardiomegaly, effusion, pneumonia, pneumothorax, and no finding. Two board-certified radiologists, each with over five years of experience, independently reviewed all of the chest X-ray images. Using the web-based annotation tool Label Studio (version 1.7) [23], they verified the presence of four different type of abnormalities for each X-ray image. Some images in the dataset were discarded as they contained multi-labels from the radiologists, such as “Effusion Pneumothorax”, in order to avoid confusion while training the model. Therefore, a total of 16,207 frontal chest X-ray images were selected to be part of this investigation. To ensure the ethical handling of sensitive data about real individuals, we obtained approval from the Institutional Review Board (IRB) [24]. This approval aligns with our commitment to respect patient autonomy and safeguard private information. As part of our data organization process, the dataset was structured into folders representing four distinct classes and one regular no finding class. Each grayscale image in the data obtained from KAAUH comprised 224 × 224 size. All the images were in good condition; however, some preprocessing steps were applied to achieve maximum results and to ensure consistency with the other public dataset, as discussed in the next section.

We have also utilized the public dataset NIH chest X-ray 14 [9]. This dataset comprises 112,120 frontal chest X-ray images, where each square image has 1024 pixels. Also, the metadata includes patient age and gender. Each image in the dataset was annotated with up to 14 of the following possible pathology labels: atelectasis, cardiomegaly, effusion, infiltration, mass, nodule, pneumonia, pneumothorax, consolidation, edema, emphysema, fibrosis, pleural thickening, and hernia. However, we chose only 4 labels, namely cardiomegaly, effusion, pneumonia, and pneumothorax, along with a no finding label, and their associated data were used to match the data obtained from KAAUH. In order to ensure consistency with the other dataset, all of the images were converted to grayscale and resized to 224 × 224. And no particular screening criteria was implemented to exclude the images. However, some preprocessing steps were applied to improve the overall quality of the images that are discussed in the next section.

The dataset exhibits substantial class imbalance, as detailed in Table 1. Some labels have high representation, such as no finding, effusion, and pneumothorax which are highly represented over the others, which posed a challenge for effective model training. The data associated with the no finding label was too high for the other classes; therefore, we only considered comparable amounts of data to other labels. Moreover, to address class imbalance issues, we applied a data augmentation technique. This method not only helped create a more balanced dataset but also improved the model’s ability to generalize, reducing the risk of overfitting and enhancing overall learning performance [25]. To enrich the dataset and improve the model’s performance, we applied several commonly used data augmentation techniques. These included random horizontal and vertical flips, rotations, cropping, and translations. Each image had a 50% chance of being flipped either horizontally or vertically. Additionally, images were randomly rotated within a range of −15 to 15 degrees and shifted up to 5% in both horizontal and vertical directions. Moreover, in some cases with low class representation, the images were subjected to zooming in and out and brightness and contrast adjustment randomly up to 10% to meet the required number of samples. These variations helped the model become more robust by learning to recognize patterns despite changes in orientation and position. The number of augmented samples varied according to their label, however, in all classes the increased number of samples were adjusted according to the number samples of the ‘No Finding’ label, as shown in Table 1.

All images from NIH chest X-ray 14 dataset were resized to 224 × 224 pixels to ensure consistency across the datasets and that they were ready to feed the training models. The data from two different sources were used in two different scenarios for training and testing to validate the generalizability of the model. In the first scenario, the NIH chest X-ray 14 dataset was used as training and the KAAUH dataset for testing. However, in the second scenario, both datasets were combined and data was then split into training and test sets using an 80:20 ratio.

### 2.2. Preprocessing of Dataset

The preprocessing step plays a critical role in the image analysis as it enhances image quality and reduces noise or irrelevant details that may reduce the effectiveness of the feature selection method or the efficiency of the classification method of the deep learning model. Therefore, in order to train the CNN-based model, the X-ray images in both datasets were processed through three different types of grayscale image enhancement methods.

In the first type of preprocessing technique, each image was normalized (*I_n_*) by subtracting each pixel of the image with its mean and dividing by its standard deviation. This step ensured that pixel values were normalized, helping the model learn more effectively.

In the second type of preprocessing technique, the histogram equalization and Gaussian blurring methods were combined (*I_g_*) to reduce image noise and suppress minor details that might confuse the neural network. A 5 × 5 kernel size was selected experimentally for the Gaussian filter. This helps in providing a good balance between noise reduction and image integrity for the neural network training.

**Figure 2:**
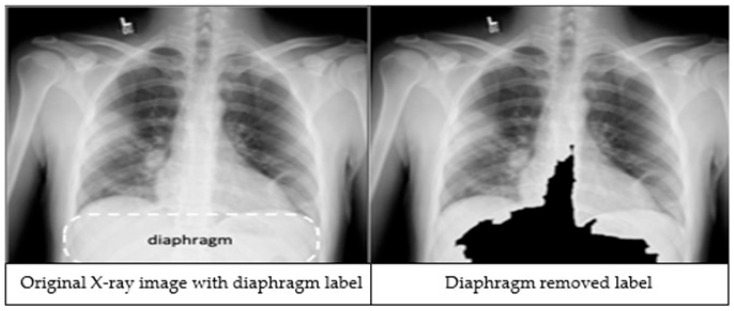
An X-ray image showing the diaphragm region of the image within the dashed lines (**left**). The image after applying the diaphragm removal technique (**right**).

The third preprocessing technique focuses on improving classification accuracy by removing the diaphragm region (see Figure 2) from the X-ray images. The process begins by identifying the maximum and minimum pixel intensities, namely *i_max_* and *i_min_* respectively. A binary threshold t = *i_min_ +* 0.9 * (*i_max_* − *i_min_*) is then applied to convert the image into a binary segmented image. Afterwards, a morphological closing is applied on the binary segmented image to make it a refined mask. Finally, a bitwise operation is performed using the mask image to remove the diaphragm area from the original image, resulting in an image which is called *I_d_*.

As the CNN-based models usually require a 3 channel (RGB) image a grayscale image of the raw dataset was replaced with three otherwise preprocessed images stacked as 3 layers, which helped in enhancing the class centric features of an image and fulfilled the input requirement of the CNN model. Now, these three types of preprocessed images (*I_n_*, *I_g_*, and *I_d_*) are stacked together, making a three channels image (224 × 224 × 3) ready for CNN-based model training.

### 2.3. Training Models

A major challenge in medical imaging research is the limited availability of annotated datasets as deep learning models typically require large volumes of labeled data, which are costly and time-intensive to obtain. Pre-trained deep learning models, a widely accepted technique, covers this limitation by leveraging knowledge from models pre-trained on large datasets to enhance feature extraction on smaller, domain-specific datasets. In this study, CNN-based models were developed using the ResNet [18], ResNext [17], and FSRFNet [22] architectures.

#### 2.3.1. RestNet50 Model

ResNet50 [18] is a specific variant of the ResNet architecture, which stands for Residual Network. ResNets were introduced to tackle the vanishing gradient problem in training deep neural networks. When the network depth increases, the gradients of the loss function become exceedingly small, leading to minimal updates in the weights of early layers and a potential halt in their learning process. The critical component in ResNet is the residual blocks, which enables the network to learn the residual, or the difference, between the input and output of a layer. The ‘50’ in ResNet50 refers to the total number of layers in the network, which is 50. A pre-trained ResNet50 was trained on over a million images from the ImageNet database.

A ResNet50 is a complex architecture, as shown in Table 2. The first block comprises three layers, followed by four layers in the second block, six layers in the third block, and concluding with three layers in the fourth block. Throughout the architecture, ResNet50 incorporates down-sampling techniques, achieved through a combination of convolutional layers with a stride more significant than one and through pooling layers. This down-sampling is executed to reduce the spatial dimensions of the feature maps while concurrently increasing channel depth. The final layers typically include global average pooling to reduce spatial dimensions to 1 × 1 before feeding into fully connected layers for classification purposes.

The idea behind ResNet is to learn the residual function F(x) of the layers rather than trying to learn the entire mapping function (H). This is achieved using residual blocks. These blocks act as a stack of convolutional layers, and the output of the block is additionally connected with its input through an identity mapping path (shortcut connection).

**Figure 3:**
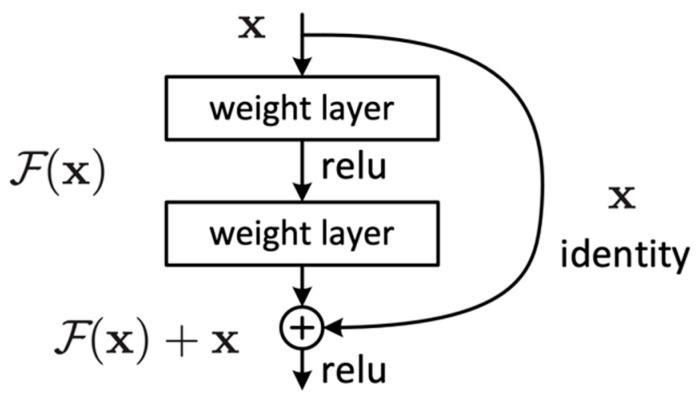
Residual block.

The residual blocks (Figure 3) are designed to allow the network to learn a residual function and then add this residual to the original input to obtain the final output. This is in contrast to conventional neural networks that aim to learn the mapping function H  that maps x to the desired output y. It can be expressed as follows:

y=H(x)

Residual blocks employ two primary paths: the identity path and the residual path. The identity path serves as a shortcut for the input, allowing it to bypass weight layers within the block and be added in the final processing stages. Meanwhile, the residual path is a direct route computing the *F*(*x*) residual mapping function. Consequently, the final process of the block is a residual function *H*(*x*) that adds up the mapping function *F*(*x*) with the input *x*, which can be expressed as follows:

(1) $$H\left(x\right)=F\left(x\right)+x$$

The ResNet learns to adjust the input such that the desired output $H\left(x\right)$ can be reached by adding the residual $F\left(x\right)$ to the input x.

#### 2.3.2. ResNext50 Model

ResNext [17] is a convolutional neural network (CNN) architecture that extends the principles of ResNet by introducing a new dimension, cardinality, or the number of parallel paths within each layer. Unlike ResNet, which employs a single transformation path with residual connections, ResNext aggregates multiple parallel transformations of the same topology within each block, enhancing the network’s capacity to learn diverse and complex features. Each cardinality group consists of multiple bottleneck blocks, typically composed of a 1 × 1 convolution for dimensionality reduction, a 3 × 3 convolution for feature extraction, and another 1 × 1 convolution for projection (Table 2). The outputs of these parallel paths are summed to form the final output of the group.

This architecture allows for simultaneous increases in both depth and width, offering flexibility in balancing computational cost and model performance. ResNext models are denoted as ResNextZ (C × 4), where C is the cardinality, C × 4 refers to the number of channels per group, and Z indicates the depth (e.g., 50 or 101 layers). By leveraging aggregated transformations, ResNext achieves improved efficiency across a variety of image-based classification tasks [26]. In this study we used ResNext50 (32 × 4), meaning a ResNext architecture-based deep learning model with a cardinality of 32, 4 channels against every group, and a depth of 50.

#### 2.3.3. FSRFNet50 Model

**Figure 4:**
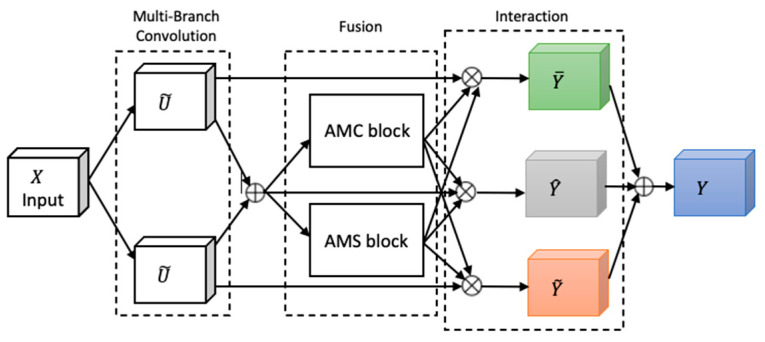
The architecture of the FSRF block [22].

Attention, a key mechanism in human perception, enables selective focus on relevant stimuli while suppressing distractions [27], and has become a central concept in cognitive neuroscience. To emulate this in neural networks, we adapted FSRFNet [22] containing a novel architectural unit—the Feature-selective and Spatial Receptive Fields (FSRF) block—to adaptively adjust receptive field (RF) sizes via the combined effects of feature-selective and spatial attention (Figure 4). The FSRF block comprises the following three operations: multi-branch convolution, fusion, and interaction between FS and RF attention.

Multi-branch convolution provides diverse filters across branches, enriching feature representation. The fusion step adaptively regulates RF sizes using gated mechanisms derived from average–max channel (AMC) and average–max spatial (AMS) attention modules, controlling the flow of multi-scale information to the subsequent layer. The AMC attention module captures inter-channel dependencies to generate a channel attention map, while the AMS module captures inter-spatial relationships to produce a spatial attention map. In the end, the information captured by the AMC and AMS modules is processed through a soft attention mechanism, enabling effective interaction between feature-selective and spatial attention.

An FSRF network (FSRFNet) is constructed by stacking multiple FSRF blocks, forming modular units referred to as FSRF units, similar to the bottleneck design in ResNext [17]. Each unit comprises a 1 × 1 convolution, an FSRF block, and another 1 × 1 convolution. In this design, conventional large-kernel convolutions are replaced by the FSRF blocks. The FSRF50 architecture, detailed in Table 2, adopts a four-stage configuration with {3, 4, 6, 3} FSRF units.

Moreover, the configuration of each FSRF block is influenced by two key hyperparameters: the cardinality of each path and the reduction ratio which controls the parameter reduction in the control fuse operator. For this experiment, we set the reduction ratio to 16 and the cardinality to 32, as discussed in [22].

### 2.4. Grad CAM

Grad CAM (Gradient-weighted Class Activation Mapping) is the technique that provides visual explanations for the activation regions of a CNN [20]. It computes gradients of the target class concerning the final convolutional layer, applies global average pooling to reduce dimensions, and then multiplies each feature map by its corresponding global average pooling weight and sums them up. The result is passed through a ReLU activation to emphasize positive importance, generating a heatmap highlighting important regions in an input image. This heatmap is then overlaid on the original image, allowing users to understand which parts of the image contribute most to the CNN’s prediction.

### 2.5. Report Generation

The report generation module employs a method that utilizes if–else statements to craft concise, descriptive sentences corresponding to each label. This approach enables the generation of clear and informative summaries tailored to the specific characteristics of the labels. The text that is used took inspiration from samples of the reports in the VinDr-CXR dataset [28] and the actual KAAUH dataset, as seen in Table 3.

## 3. Results

In this section, we will discuss the experimental details and obtained results. In this study, pre-trained ResNet50, ResNext50 and FSRFNet50 models were utilized, leveraging their prior training on large-scale datasets such as ImageNet. The final layer of all the CNN-based models was replaced with the softmax layer with five classes. This layer calculates the probability distribution over all the classes by utilizing the features extracted from preceding layers. Henceforth, a particular class with the highest probability is selected as a predicted label by the network. To enhance model performance, hyperparameter optimization is conducted by adjusting key parameters, including the learning rate (0.001), optimizer (Adam with weight decay of 0.001), and dropout rate. The grid search technique was employed as optimization technique to identify the most effective configuration based on validation performance.

The proposed RadAI model is trained and evaluated using chest X-ray images. This experimental study utilized an Intel i7 processor and an NVIDIA GeForce GTX3060 graphics card. The training dataset of both scenarios is split into a 4:1 ratio for training and validation, respectively, and fivefold cross-validation is utilized to ensure robustness of the training model. For training the model, we used a batch size of 32 over 100 epochs. The Adam optimizer was chosen for its efficiency and reliability in handling deep learning tasks. The cross-entropy was used as loss function. An early stopping mechanism was utilized to prevent overfitting. Additionally, the experiments were conducted three times to ensure that the models were independent of the learning data. It should be noted that no preprocessed images were used for testing purposes. The model’s performance is assessed using multiple classification metrics, including accuracy, recall, precision, and F1-score.

The results obtained using the proposed RadAI model have been presented in Table 4, Table 5, Table 6 and Table 7. The proposed method with the FSRFNet50 model achieved accuracies of over 97% using dataset scenarios 1 and 2. Even without combining the datasets, all the training models have shown satisfactory performance on limited data. Moreover, the preprocessing technique has raised the precision, recall, and F1-score by about 3% on average. The results validate that RadAI has improved the abnormality detecting efficiency using chest X-ray images. Additionally, using separate datasets for training and testing purposes has made the CNN models unbiased. The ResNext50 performed more effectively over ResNet50 due to the advanced feature extraction mechanism.

**Figure 5:**
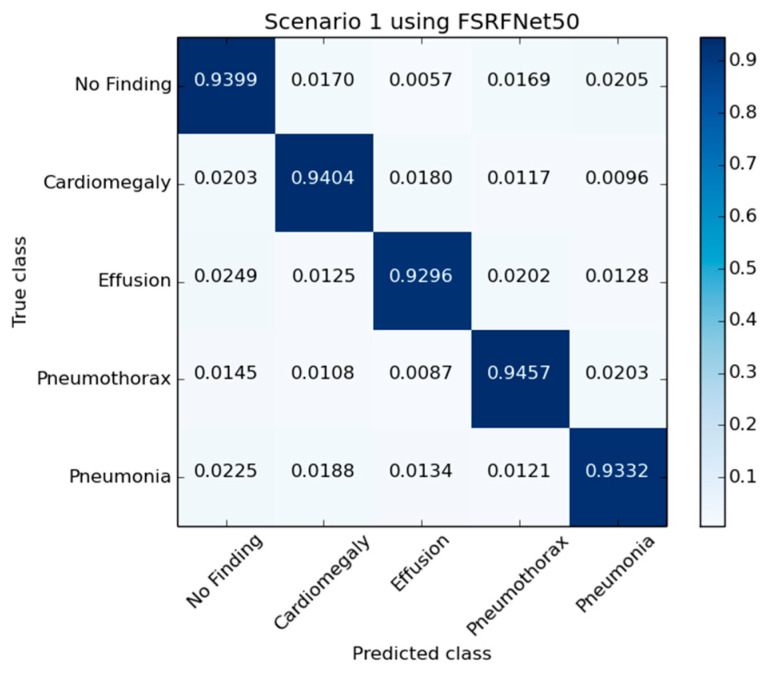
The confusion matrix of RadAI using FSRFNet50 on scenario 1.

**Figure 6:**
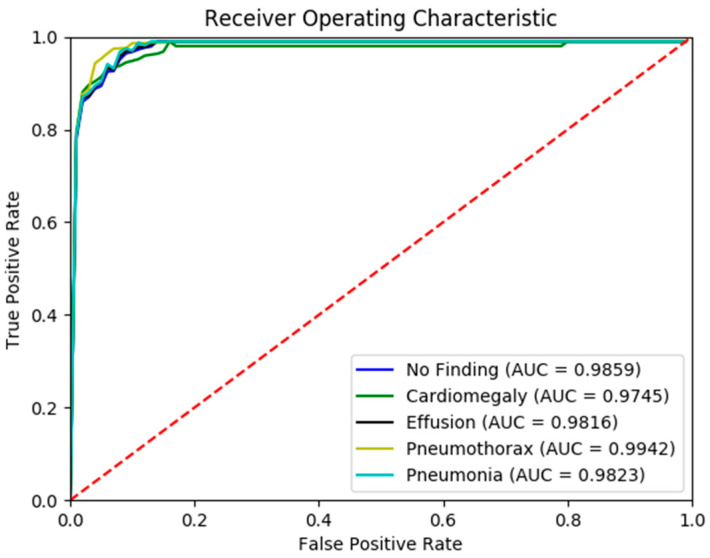
The ROC curve of RadAI using FSRFNet50 on scenario 1.

Figure 5 presents the confusion matrix generated by the FSRFNet50 model, illustrating its classification performance. Additionally, the receiver operating characteristic (ROC) (Figure 6) analysis indicates that the model achieved the highest area under the curve (AUC) score of 0.9942 for the pneumothorax class, demonstrating excellent discriminative capability for this pathology.

**Figure 7:**
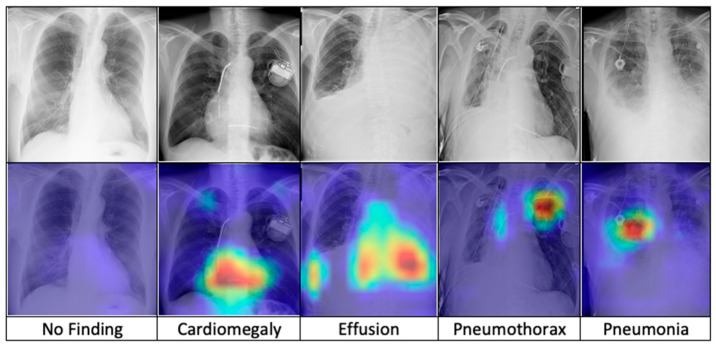
The comparison of heatmaps among five categories of chest X-ray images selected for classification.

After training, the model is combined with the Grad-CAM algorithm to generate saliency maps for the test images. These maps are overlaid on the original chest X-ray images to visualize class-discriminative regions, highlighting the areas that are most influential in predicting the five diagnostic categories. Figure 7 presents a comparative visualization of the resulting heatmaps across cardiomegaly, effusion, pneumothorax, pneumonia, and no finding.

A comparison of the proposed model using the open-source dataset NIH chest X-ray 14 has also been provided in Table 8. It is evident that the proposed model with the FSRFNet50 model has outperformed the studies from the literature in terms of the abnormalities under discussion.

## 4. Discussion

We explore the outcomes derived from the deployment of three advanced deep learning models—ResNet50 [18], ResNext50 [17], and FSRFNet50 [22]—implemented for classifying abnormalities in chest X-ray images. By integrating these findings within the existing literature, the discussion aims to underline the technological advances and clinical implications of each model. Highlighting the contributions of this research, the aim is to showcase the potential enhancements in diagnostic accuracy and reliability that these models bring to the field of radiology.

The experimental results reveal that, while using the KAAUH dataset along with NIH chest X-ray 14 dataset, the FSRFNet50 model demonstrated superior performance metrics in comparison to ResNet50 and ResNext50. The model achieved over 97% accuracy, showing its ability to differentiate between four rudimentary chest conditions. The higher accuracy and a more balanced precision–recall trade-off signifies its robustness and reliability for X-ray images.

We applied image preprocessing techniques to automatically detect and remove the diaphragm region from chest X-ray images. Comparative analysis revealed that excluding the diaphragm significantly improved classification accuracy, increasing from 92.69% to 97.29%. While deep learning models are typically capable of learning relevant features without explicit segmentation, our findings demonstrate that removing irrelevant regions through preprocessing can enhance both the performance and robustness of the models.

The superior performance of the FSRFNet50 model can be primarily attributed to its sophisticated architectural design, which efficiently handles the inherent variability of X-ray imaging data. While high performance metrics are important, model interpretability is equally critical in medical applications. To enhance transparency, we employed Grad-CAM to visualize the regions of X-ray images that the model emphasizes during prediction. These visualizations offer insights into the model’s decision-making process, supporting both clinical relevance and model refinement. Our experiments showed that incorporating interpretability techniques not only improved understanding but also contributed to optimizing model accuracy. As seen in Figure 7 (from left to right), the Grad-CAM heatmaps demonstrate the interpretability of the proposed RadAI model across various thoracic pathologies. For normal cases, no significant regions are activated, aligning with the “no finding” prediction. In cases of cardiomegaly, the heatmap highlights the enlarged heart region, while pleural effusion is accurately localized in the corresponding X-ray images. For pneumothorax, strong activation is observed in the left lung area. Although pneumonia cases are more complex due to diffuse patterns, the heatmap still reveals high-probability regions, supporting the model’s classification. These visualizations not only validate the model’s ability to localize disease-relevant features effectively, but also help the radiologists to analyze the activated region effectively.

Nevertheless, it is crucial to acknowledge the difficulties and challenges faced while improving and finalizing the model. One difficulty was dealing with the class imbalance and how it affected the accuracy. We incorporated an augmentation process which compensated the class-imbalance problem but made the model prone to overfitting. Although this study discussed the improvement of image quality by preprocessing steps, it needs more refinement, and optimal techniques need to be investigated. Moreover, the accuracy of X-ray image-based model outcomes are influenced by factors such as image quality, resolution, and the size or stage of the X-ray image. Moreover, CNN-based architectures typically require large, well-annotated datasets for effective training and validation to ensure robust and reliable performance in practical medical image analysis.

Several dataset-related challenges emerged during the analysis of chest X-ray data. To maintain consistency, multi-labeled images were excluded as multiple labels per image introduced irregularities that could hinder model training. Additionally, some images were rotated or contained artifacts such as foreign objects and clothing, introducing noise into the training process. Although these artifacts were not part of the exclusion criteria—reflecting real-world radiological scenarios—they negatively affected model performance. Unlike prior work by Tang et al. [32], this study was constrained by the use of a single GPU, limiting model complexity to ResNet50 instead of deeper variants like ResNet101 or ResNet151, which may enhance future performance. Despite these limitations, the model produced promising results. Combining expert domain knowledge with advanced deep learning models could further improve diagnostic accuracy, efficiency, and cost-effectiveness in medical imaging. Nonetheless, for AI systems to play a more autonomous role in clinical settings their decisions must closely align with human-validated ground truth.

The results of this study also highlight the critical role of data diversity and volume in training robust CNN-based models. Trained on more comprehensive datasets, CNN-based models exhibited improved generalizability and performance, suggesting that future implementations should focus on expanding and diversifying training datasets to reflect the complexity of real-world scenarios better. The findings from this research make significant contributions to the advancement of medical imaging technology, particularly through the enhancement of automated chest X-ray analysis. The study not only demonstrates the potential of deep learning models to enhance diagnostic efficiency but also emphasizes the importance of model interpretability and the need for extensive dataset variability.

## 5. Conclusions

This study aimed to automate a critical stage of assessing radiology images by employing three different models, namely FSRFNet50, ResNext50 and ResNet50, to accurately detect four abnormalities and a “no finding” category from chest radiographs. The models were trained and evaluated using the NIH chest X-ray 14 dataset and a private dataset from KAAUH, both comprising diverse abnormalities. Model performance was assessed based on their ability to predict the presence of specific diseases. In conclusion, after comparing the three architectures on two datasets in terms of accuracy, precision, recall, and F1-score, the FSRFNet50 consistently attained better precision, highlighting how good the model architecture is regardless of the dataset used.

In the future, the RadAI project can expand to developing a web portal and a mobile application for the detection of chest X-ray images to assist radiologists in remote areas. Moreover, beyond chest X-ray diagnosis, RadAI may include the analysis of X-rays from various organs using deep learning models in the future. This expansion would provide doctors with a comprehensive view of a patient’s medical condition by allowing them to review X-rays from all organs. Additionally, the project could seek to develop a system that not only assists in the analysis but also generates detailed reports based on the observations made by the practicing medical experts. This integration of multi-organ X-ray analysis and report generation has the potential to improve diagnostic accuracy, efficiency, and overall patient care in the future.

## Figures and Tables

**Table 1:** Datasets statistics with respect to selected labels + augmented data.

Labels	KAAUH Dataset	NIH Chest X-Ray 14 Dataset	Combined
No Finding	5600 + 0	20,000 + 0	25,600 + 0
Cardiomegaly	1668 + 3932	2776 + 17,224	4444 + 21,156
Effusion	4800 + 800	13,317 + 6683	18,117 + 7483
Pneumothorax	3239 + 2361	5302 + 14,698	8541 + 17,059
Pneumonia	900 + 4700	1431 + 18,569	2331 + 23,269
Total	16,207 + 11,793	83,187 + 57,174	99,394 + 68,967

**Table 2:** Architectures for ResNet [18], ResNext [17], and FSRFNet [22]. Building blocks are shown in brackets with the number of blocks stacked.

Stage	Output	ResNet50	ResNext50 (32 × 4d)	FSRFNet50
Conv1	112×112	7×7, 64, stride 2	7×7, 64, stride 2	7×7, 64, stride 2
Conv2	56×56	3×3 max pool, stride 2	3×3 max pool, stride 2	3×3 max pool, stride 2
		$\left[\begin{array}{c} 1\times 1,64\\ 3\times 3,64\\ 1\times 1,256 \end{array}\right]\times 3$	$\left[\begin{array}{c} 1\times 1,128\\ 3\times 3,128\\ 1\times 1,256 \end{array},\;C=32\right]\times 3$	$\left[FSRF\left[\begin{array}{c} 1\times 1,128\\ M=2,G=32,r=16\\ 1\times 1,512 \end{array}\right],128\right]\times 3$
Conv3	28×28	$\left[\begin{array}{c} 1\times 1,128\\ 3\times 3,128\\ 1\times 1,512 \end{array}\right]\times 4$	$\left[\begin{array}{c} 1\times 1,256\\ 3\times 3,256\\ 1\times 1,512 \end{array},\;C=32\right]\times 4$	$\left[FSRF\left[\begin{array}{c} 1\times 1,256\\ M=2,G=32,r=16\\ 1\times 1,512 \end{array}\right],256\right]\times 4$
Conv4	14×14	$\left[\begin{array}{c} 1\times 1,256\\ 3\times 3,256\\ 1\times 1,1024 \end{array}\right]\times 6$	$\left[\begin{array}{c} 1\times 1,512\\ 3\times 3,512\\ 1\times 1,1024 \end{array},\;C=32\right]\times 6$	$\left[FSRF\left[\begin{array}{c} 1\times 1,512\\ M=2,G=32,r=16\\ 1\times 1,1028 \end{array}\right],512\right]\times 6$
Conv5	7×7	$\left[\begin{array}{c} 1\times 1,512\\ 3\times 3,512\\ 1\times 1,2048 \end{array}\right]\times 3$	$\left[\begin{array}{c} 1\times 1,1024\\ 3\times 3,1024\\ 1\times 1,2048 \end{array},\;C=32\right]\times 3$	$\left[FSRF\left[\begin{array}{c} 1\times 1,1024\\ M=2,G=32,r=16\\ 1\times 1,2048 \end{array}\right],1024\right]\times 3$
	1×1	global average pool 1000-d fc, softmax	global average pool 1000-d fc, softmax	global average pool 1000-d fc, softmax

**Table 3:** Labels along with their sample reports.

Label	Sample Report
No Finding	The imaging examination of the chest revealed no abnormalities. The patient’s heart size and shape appear normal, and there is no evidence of fluid around the lungs or pleura (lining around the lungs and chest wall).
Cardiomegaly	Cardiomegaly is present. The heart appears enlarged on the X-ray. No pleural effusion, pneumonia, or pneumothorax were identified.
Effusion	The examination shows fluid accumulation in the space around the lungs (pleural effusion). Various factors, including infection, heart failure, or liver disease, could cause this. The patient’s heart size appears normal.
Pneumonia	The examination reveals signs of infection within the lung tissue, consistent with pneumonia. This may present symptoms like cough, fever, and shortness of breath. The patient’s heart size and surrounding area appear normal.
Pneumothorax	The imaging shows air present in the space between the chest wall and the lung (pneumothorax). This can cause sudden chest pain and shortness of breath. The patient’s heart size appears normal, and there is no significant pleural effusion.

**Table 4:** Summary of performance of RadAI using predefined scenarios.

Scenario	Models	Accuracy	Recall	Precision	F1-Score
Scenario 1	ResNet50	94.42	93.71	92.79	93.53
	ResNext50	95.31	94.94	94.56	95.26
	FSRFNet50	**97.29**	97.23	96.77	96.81
Scenario 2	ResNet50	95.37	95.22	95.07	95.12
	ResNext50	95.96	95.12	95.13	94.22
	FSRFNet50	**97.25**	96.55	96.68	97.15

**Table 5:** Summary of performance of RadAI according to individual classes using FSRFNet50.

Scenario	Classes	Accuracy	Recall	Precision	F1-Score
Scenario 1	No Finding	97.45	97.78	96.59	96.94
	Cardiomegaly	96.82	96.99	97.04	96.94
	Effusion	97.16	96.94	96.75	97.48
	Pneumothorax	**97.67**	96.86	96.54	96.20
	Pneumonia	97.34	97.58	96.96	96.53
Scenario 2	No Finding	**97.56**	95.96	97.85	97.76
	Cardiomegaly	97.23	96.45	96.65	97.01
	Effusion	97.28	96.68	96.01	96.63
	Pneumothorax	96.85	96.77	96.33	96.88
	Pneumonia	97.31	96.88	96.56	97.50

**Table 6:** Summary of performance of RadAI using individual datasets.

Dataset	Models	Accuracy	Recall	Precision	F1-Score
NIH Chest X-ray 14	ResNet50	93.18	92.54	92.38	92.38
	ResNext50	93.89	93.58	93.68	92.97
	FSRFNet50	**94.05**	93.48	92.97	93.01
KAAUH	ResNet50	92.01	91.91	91.63	91.67
	ResNext50	92.65	92.80	91.93	92.03
	FSRFNet50	**93.25**	93.09	92.71	92.56

**Table 7:** Results obtained without preprocessing using FSRFNet50 model on individual dataset.

Dataset	Models	Accuracy	Recall	Precision	F1-Score
NIH Chest X-ray 14	No Finding	92.09	92.49	92.3	91.93
	Cardiomegaly	**93.81**	93.52	92.39	93.41
	Effusion	92.42	91.91	92.18	93.21
	Pneumothorax	92.72	91.55	91.14	92.27
	Pneumonia	92.43	92.23	92.08	91.97
KAAUH	No Finding	89.28	88.04	88.93	89.32
	Cardiomegaly	90.03	88.03	89.84	87.78
	Effusion	89.59	88.59	87.22	85.29
	Pneumothorax	**91.64**	90.71	91.35	91.76
	Pneumonia	89.79	87.92	89.73	88.81

**Table 8:** The comparison of proposed RadAI results with state-of-the-art models in the literature with the NIH chest X-ray 14 dataset only.

Reference	Model	No Finding	Cardiomegaly	Effusion	Pneumothorax	Pneumonia
Mann et al. [11]	CheXNet	-	0.9248	0.8638	0.8887	0.7680
	DenseNet121	-	0.9120	0.8830	0.8670	0.7430
	ResNet50	-	0.74.10	0.8600	0.7790	0.6940
	EfficientNetB1	-	0.8840	0.8460	0.8100	0.7130
Kufel et al. [29]	EfficientNet	-	0.911	0.879	0.898	0.769
Hage Chehade et al. [30]	DenseNet-121 with CycleGAN	0.8496	0.9358	0.9036	9138	0.8387
Nawaz et al. [31]	EfficientNet-B0 with BiFPN	-	0.95	0.93	0.91	0.84
Proposed	ResNet50	0.8921	0.8992	0.9048	0.9121	0.8963
	ResNext50	0.9041	0.9137	0.9104	0.9184	0.9016
	FSRFNet50	**0.9209**	**0.9381**	**0.9242**	**0.9272**	**0.9243**

## Data Availability

Data is available from the authors upon reasonable request.
